# Applying neutral drift to the directed molecular evolution of a β-glucuronidase into a β-galactosidase: Two different evolutionary pathways lead to the same variant

**DOI:** 10.1186/1756-0500-4-138

**Published:** 2011-05-06

**Authors:** Wendy S Smith, Jennifer R Hale, Cameron Neylon

**Affiliations:** 1 School of Chemistry, University of Southampton, Highfield SO17 1BJ, UK; 2 Science and Technologies Facilities Council, Rutherford Appleton Laboratory, Didcot, OX11 0QX, UK

## Abstract

**Background:**

Directed protein evolution has been used to modify protein activity and research has been carried out to enhance the production of high quality mutant libraries. Many theoretical approaches suggest that allowing a population to undergo neutral selection may be valuable in directed evolution experiments.

**Findings:**

Here we report on an investigation into the value of neutral selection in a classical model system for directed evolution, the conversion of the *E. coli *β-glucuronidase to a β-galactosidase activity. We find that neutral selection, i.e. selection for retaining glucuronidase activity, can efficiently identify the majority of sites of mutation that have been identified as beneficial for galactosidase activity in previous experiments. Each variant demonstrating increased galactosidase activity identified by our neutral drift experiments contained a mutation at one of four sites, T509, S557, N566 or W529. All of these sites have previously been identified using direct selection for beta galactosidase activity.

**Conclusions:**

Our results are consistent with others that show that a neutral selection approach can be effective in selecting improved variants. However, we interpret our results to show that neutral selection is, in this case, not a more efficient approach than conventional directed evolution approaches. However, the neutral approach is likely to be beneficial when the resulting library can be screened for a range of related activities. More detailed statistical studies to resolve the apparent differences between this system and others are likely to be a fruitful avenue for future research.

## Background

The development of experimental approaches for directed evolution has mirrored, to a certain extent, the growing sophistication of our understanding of natural evolution. Early experiments applied mutagenesis at random along the length of a protein coding gene followed by the application of a direct selection pressure [1,2], an approach that Darwin would have readily understood. Directed protein evolution has been used successfully to tailor protein properties and to advance the understanding of structure function relationships. However, despite advances in screening technologies only a small fraction of theoretical protein sequence can be sampled in experiments. Recently new theoretical concepts and computational programmes have been developed with the aim of producing high quality mutant libraries [3]. Advances in random and focussed mutagenic techniques such as the trinucleotide exchange method (TriNEx) [4], transversion enriched sequence saturation mutagenesis (SeSaM-Tv) [5] and iterative saturation mutagenesis (ISM) [6] may aid the generation of libraries with functionally enriched diversity.

In natural systems neutral drift is also likely to play a role in priming a population for evolution. Neutral drift is the incorporation of mutations which have little or no effect on the protein in its current environment. Neutral mutations however, may have adaptive potential and allow adaptive events to occur more frequently. Adaptive mutations in isolation are rare [7,8] as many mutations are potentially deleterious, undermining protein stability or folding. These adaptive mutations which are deleterious in isolation may however be able to be included if other neutral mutations are first introduced into the gene; or through the introduction of stabilising mutations, which can bring residues into line with residues shared by the common ancestor or the consensus residue across a protein family [9,10]. It is thought that acquisition of new protein functions occurs in steps produced by single beneficial mutations, with the potential for the initial steps to occur before selection for the new function [8,10-12].

This poses the question; is it possible to modify a protein's activity using neutral drift and produce variants with higher activity towards an alternative substrate than previously demonstrated using direct selection experiments?

Bloom *et al*. [8] investigated how cytochrome P450 enzymes that have evolved neutrally with respect to activity on a single substrate changed in their abilities to catalyze reactions on other substrates. They concluded that neutral genetic drift can lead to substantial changes in protein functions that are not currently under selection, preparing the proteins to more readily undergo functional evolution should selection favour new functions in the future.

In a classic directed evolution experiment Matsumura and Ellington [13] converted the *E. Coli *β-glucuronidase towards β-galactosidase activity in three rounds of mutagenesis and recombination. Their best variant contained four mutations and further extensive screening of libraries generated by random mutagenesis failed to identify any variants with further improvements in activity. We have carried out an equivalent directed evolution experiment introducing neutral selection in an attempt to identify other variants with further improvements to activity through random mutagenesis.

## Results

### Screening system for neutral selection

It was necessary to identify an E. coli strain with no endogenous glucuronidase or galactosidase activity so that both activities of interest could be screened in the same cells. We selected BW25141 cells [14] (gusA-, lacZ-) for this purpose. We found that this strain was much more efficiently transformed with purified plasmid than with the products of ligation reactions and elected to use a two stage approach. The wild type gusA gene was subjected to random mutagenesis using error-prone PCR or the Stratagene Genemorph system. The library of PCR products was ligated between the NcoI and EcoRI sites of pBAD/His (Invitrogen) and transformed into super competent XL1-Blue cells (Stratagene) to generate a large number of colonies (6 000 - 15 000 in experiment A). The colonies were re-suspended in LB medium and plasmid DNA was isolated. The purified plasmid DNA was then used to transform BW25141 cells generating 800 - 3 000 colonies (experiment A). Glucuronidase positive colonies, where glucuronidase activity is defined as a colony displaying intense blue colouration, were selected on plates containing saturating concentrations of X-glu (5 bromo-4-chloro-3-indolyl-β-Dgalactoside) and 2 mg.mL^-1 ^arabinose. See [Additional file 1: Supplementary Figure S1] for an indication of colour variation.

### Optimisation of mutation rate

The most common 'variant' in a library that has been screened for maintenance of function will be the wild-type protein. In initial experiments with a low mutagenesis rate we recovered entirely wild-type glucuronidase genes from neutral screens. At much higher mutagenesis rates the number of blue (neutral) variants dropped to less than 10% which would lead to very low diversity in the neutral library. The mutation rate was therefore optimised by varying the concentration of input DNA in the mutagenic PCR from 16 - 125 ng which gave percentages of white colonies varying from 50 - 30% respectively. The concentration of target DNA used in the preparation of the libraries was 31 ng in experiment A and 16 ng in experiment B corresponding to mutagenesis rates of one to two substitutions per gene per round of screening.

### Selection of galactosidase positive variants

**Figure 1:**
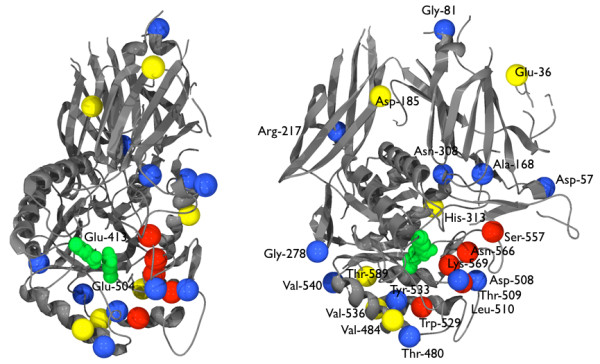
**Position of neutral mutations in the *E. coli *β-glucuronidase**. The *gusA *gene was subjected to four rounds of neutral screening in which 1815, 801, 1341, and 952 blue (*gusA*^+^) colonies were selected respectively. The fourth round neutral library was then screened for β-galactosidase activity. The five residues where mutations contributed to galactosidase activity identified in this study and previously are shown in red. Neutral mutations identified in the neutral screen are shown in yellow. Mutations that were identified in the galactosidase screen but did not contribute significantly to galactosidase activity are shown in blue. The *E. coli *β-glucuronidase sequence was modelled on to the human glucuronidase structure [26], using Swiss-Model [27,28].

Four rounds of neutral selection were carried out on the *gusA *gene using 31 ng of template DNA in each round of PCR based random mutagenesis (Experiment A). Six glucuronidase positive variants from the fourth round were chosen at random, and plasmid DNA was isolated and sequenced. The neutral mutations identified were E36K, D185G, P266S, H313Y, V484I, V536I, T589I, and G594D. The positions of these mutations in a modelled structure of the β-glucuronidase are shown in Figure 1 in yellow. After four rounds of neutral selection (see [Additional file 2: Supplementary Table T1] for colony numbers), in which a total of around 8000 colonies and an estimated 24 000 mutations were screened, the resulting neutral library was screened for β-galactosidase activity. After 24 hours of incubation on plates containing arabinose and 5 bromo-4chloro-3-indolyl-β-D-galactoside (x-gal) those colonies appearing bluer than control colonies expressing the wild type β-glucuronidase were picked, and plasmid DNA was isolated and sequenced. Three unique gene sequences were identified and the substitutions found are detailed in Table 1. In every galactosidase positive gene sequenced a substitution was found at one of two sites, T509 and N566. These are sites previously identified by Matsumura and Ellington [13] in their direct selection experiments. These single substitutions were introduced into wild type *gusA *using a Stratagene site directed mutagenesis kit and confirmed by DNA sequencing. Plasmids containing genes for proteins with single residue substitutions as well as the plasmids isolated from the screening experiment were transformed into BW25141 cells and screened for β galactosidase activity. There was no significant difference in intensity between N566S and L510F/N566S. L510F alone was much paler. T509A colonies produced a similar blue colour to colonies of G278A/N308D/T509A. Thus the β-galactosidase activity appears to be due to single key mutations, more specifically mutations that have been identified in previous direct selection experiments. See [Additional file 2: Supplementary Figure S1] for an indication of colour variation.

**Table 1:** Sequences of variants exhibiting β-galactosidase activity.

	Wild Type	A 4.1	A 4.3	A 4.5	A 5.1	A 5.2	B 4.1	C 4.1	C 4.2	Neutral 1	Neutral 2	Shuffled-1	Shuffled-2
AA substitutions		2	3	4	4	3	2	3	3	2	5		

Silent Mutations		2	1	1	3	0	1						

													

Position													

36	E										K		

42	S											N	

57	D				N								

67	V								D				

81	G					V							V

185	D										G		

168	A					S							

217	R			C									

245	G							A					

266	P										S		

278	G		A										

308	N		D										

313	H										Y		

367	A			P									

370	K							R	R				

378	E											R	

480	T						A						

484	V									I			

498	Q												R

508	D				G								

509	T		A	A								A	A

510	L	F											

529	W					L		L	L				

533	Y				C								

536	V									I			

540	V			D									

557	S				A							P	P

566	N	S					S					S	S

568	K											Q	Q

594	G										D		

Variants A4.1 - 4.5 were obtained from experiment A after four rounds of neutral screening. Variants A5.1 and A5.2 were obtained from the fifth round of neutral screening. Variant B4.1 was obtained from experiment B using a high mutagenesis rate, after four rounds of neutral screening. Variants C4.1 and C4.2 were obtained from experiment C after four rounds of neutral screening. Shuffled-1 and Shuffled-2 are the variants obtained by DNA shuffling of the variants with galactosidase activity. The positions where mutations were previously identified by Matsumura and Ellington [13] are highlighted.

Transformants from the fourth round of neutral mutagenesis were also screened for glucuronidase activity, 952 deep blue colonies were selected, and subjected to a further round of mutagenic PCR. The resulting library was screened for β-galactosidase activity. Two variants showing galactosidase activity were identified; D57N/D508G/Y533C/S557A and G81V/A168S/W529L. The D508 and S557 sites are two other mutations identified by Matsumura and Ellington [13]. The W529L mutation was identified in a direct selection experiment, using a solution based screening system, by Rowe *et al*. [15].

In experiment B 16 ng of target DNA was used in the error prone PCR reactions to give a higher mutation rate. After four rounds of neutral selection (see [Additional file 3: Supplementary Table T1] for colony numbers) and screening for galactosidase activity a single unique galactosidase positive variant was identified. The plasmid isolated coded for a glucuronidase enzyme with T480A and N566S substitutions. The N566S substitution was identified in the first experiment and by Matsumura and Ellington [13].

The Genemorph II™ random mutagenesis kit, combining both Mutazyme and *Taq*, was used in experiment C, thus eliminating the mutational bias encountered using each enzyme alone. Screening for β-galactosidase activity after four rounds of neutral selection provided two variants; G245A/K370R/W529L and V67D/K370R/W529L. The W529L mutation was again present. K370 has not been identified previously as advantageous for increasing β-galactosidase activity but was identified as a mutation site in a direct selection experiment to increase the thermal stability of β-glucuronidase by Flores and Ellington [16].

In experiments A, B and C the number of glucuronidase positive variants taken through to the next round ranged from 800 to 3262. An experiment was carried out using smaller library sizes of less than 600 variants per round. Following screening for β-galactosidase activity no positive variants were found.

In summary each variant demonstrating increased galactosidase activity identified by our neutral drift experiments contained a mutation at one of four sites, T509, S557, N566 or W529 (shown in red in Figure 1). All of these sites have previously been identified in direct selection experiments [13,16].

### Characterisation of neutral and galactosidase positive variants

**Figure 2:**
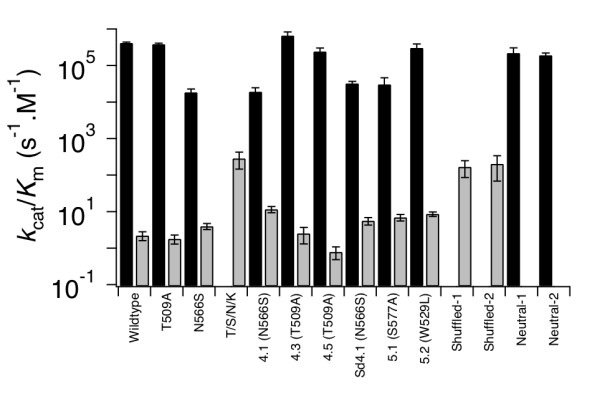
**Kinetic parameters for selected variants**. Names of the variants are as given in Table 1. Glucuronidase activity is shown with black bars, galactosidase activity with grey bars. Error bars are the standard errors from fits of the data to a Michaelis-Menten model. Variant glucuronidases were overexpressed from pBAD derived plasmids in BW25141 cells. The cells were lysed and the protein purified by Ni-NTA sepharose FF chromatography followed by concentration and buffer exchange by ultra filtration. Protein concentration was estimated by the method of Bradford [29]. Enzyme activity was quantified using the substrates *p*-nitrophenyl-β-D-galactopyranoside (pNP-gal) and *p*-nitrophenyl-β-D-glucuronide (pNP-glu) in 50 mM Tris HCl buffer pH 7.4. The absorbance at 405 nm was monitored using a Safire2 microplate reader (Tecan). An extinction coefficient of 17 400 M^-1 ^cm^-1 ^was used to calculate *p*-nitrophenyl-phosphate concentration. The concentration of protein used ranged from 0 to 200 nM in the glucuronidase assays and from 500 nM to 5 μM in the galactosidase assays. The concentration of pNP-gal ranged from 100 μM to 15 mM and that of pNP-glu varied between 10 μM and 5 mM.

Selected variants, along with the wild type β-glucuronidase, were purified and their activities were characterised in solution using *p*-nitrophenyl-β-D-galactopyranoside (pNP gal) and *p-*nitrophenyl-β-D-glucuronide (pNP glu) as substrates. The kinetic parameter kcat/Km is shown in Figure 2 and the individual parameters are shown in Table 2. The most active mutants produced from the neutral libraries were A4.1 (showing a 6 fold increase in kcat/Km) and A5.1 (four fold increase in kcat/Km) in terms of galactosidase activity. These mutants contain the substitutions N566S and S557A respectively. The N566S substitution alone produced a two fold increase in kcat/Km using p nitrophenyl β-D-galactopyranoside as a substrate, comparing well with the results obtained by Matsumura and Ellington [13].

**Table 2:** Kinetic parameters for pNP-glycoside hydrolysis by selected variant glucuronidases.

	β-glucuronidase activity			β-galactosidase activity		
Variant	*k*_cat _(s^-1^)	*K*_M _(μM)	*k*_cat_/*K*_M _(M^-1^.s^-1 ^× 10^3)^	*k*_cat _(s^-1 ^× 10^-3)^	*K*_M _(mM)	*k*_cat_/*K*_M _(M^-1^.s^-1^)

Wild-type	109 ± 42	260 ± 120	410 ± 20	6.0 ± 0.2	2.7 ± 0.7	2.2 ± 0.6

N566S	128 ± 36	7000 ± 2000	18 ± 4	28 ± 2	7.2 ± 2	3.9 ± 0.8

T509A	161 ± 30	420 ± 60	380 ± 30	10 ± 2	5.4 ± 2.9	1.8 ± 0.5

T/S/N/K	-	-	-	99 ± 18	0.35 ± 0.2	280 ± 140

A4.1	55 ± 19	2900 ± 500	19 ± 6	21 ± 5	1.8 ± 0.8	12 ± 2

A4.3	129 ± 13	200 ± 40	660 ± 180	15 ± 8	6.0 ± 0.3	2.5 ± 1.2

A4.5	58 ± 6	240 ± 30	240 ± 60	2 ± 1	2.4 ± 1.3	0.8 ± 0.3

B4.1	46 ± 8	1400 ± 300	32 ± 5	15 ± 5	2.7 ± 1.1	5.5 ± 1.3

A5.1	2.7 ± 0.8	89 ± 38	30 ± 16	148 ± 43	22 ± 5	6.8 ± 1.4

A5.2	83 ± 22	280 ± 110	300 ± 90	63 ± 12	7.3 ± 2.6	8.6 ± 1.2

Shuffled-1	-	-	-	46 ± 9	0.3 ± 0.1	166 ± 80

Shuffled-2	-	-	-	96 ± 17	0.5 ± 0.3	200 ± 134

Neutral-1	77 ± 21	35 ± 5	220 ± 90	-	-	-

Neutral-2 C4.1	83 ± 9 21.8	440 ± 80 73.6	190 ± 30 296	- 9	- 9.21	- 0.98

The parameters were estimated as described in Materials and Methods. The parameters and errors for each parameter are derived from an independent fit of the data to a Michaelis-Menten model. Glucuronidase activity for Shuffled-1, Shuffled-2, and the T/S/N/K variant, and galactosidase activity for Neutral-1 and Neutral-2 were not detectable under the conditions of our assay.

The mutant T509A and other mutants containing this residue showed no significant increase in the kcat/Km values obtained using p-nitrophenyl β-D galactopyranoside as a substrate. However, blue colonies were visible on LB amp arabinose x-gal plates. Matsumura and Ellington measured a four fold increase in activity for the mutant T509A using p-nitrophenol galactoside as a substrate [13]. The discrepancy with our results may be due to the differences in the assays used, for example, the difference in substrate leaving group. All of these experiments used purified and re-transformed plasmid. The T509A/S557P/N566S/K568Q variant, the best identified by Matsumura and Ellington, showed over a hundred fold increase in galactosidase activity compared to the wild type glucuronidase enzyme. The K568Q mutation identified by Matsumura and Ellington was not found in any of the galactosidase positive mutants selected from the neutral selection libraries. This is expected as K568Q has a deleterious effect on glucuronidase activity. Plasmids containing a gene coding for K568Q β-glucuronidase also produced white colonies when screened for glucuronidase activity. Two variants from the fourth round neutral library obtained during experiment A (neutral 1 and neutral 2) retained high β glucuronidase activity but no β galactosidase activity was measurable at the protein and substrate concentrations used, indicating less galactosidase activity than the wild type glucuronidase itself showed.

The mutants N566S, A4.1 and B4.1 produced twenty fold lower Kcat/km values than wild type β glucuronidase when *p-*nitrophenyl-β-D-glucuronide was used as a substrate. This may indicate that the selection level is relaxed. However, no significant colour variation between colonies was apparent from the × glu screening when blue colonies were selected. The x-glu/gal screen is a qualitative assay as opposed to the quantitative assay used to determine the kinetic data.

### DNA shuffling

Five variants, selected from both the fourth and fifth round neutral libraries (A4.1, A4.3, A5.1, A5.2, B4.1) obtained from experiments A and B, were pooled and subjected to DNA shuffling [17,18]. Six blue colonies were obtained when the transformants resulting from the shuffled, pooled mutants were screened for beta galactosidase activity, using x-gal, and these yielded two unique variants on sequencing; S42N/E378A/T509A/S557P/N566S/K568Q (Shuffled 1) and G81V/Q498R/T509A/S557P/N566S/K568Q (Shuffled 2). Both of these variants contain the same four substitutions as the best variant identified in the direct selection experiment. All but one of the substitutions that were present in the pooled variants but identified as unimportant above (D57N, A168S, R217C, G278A, N308D, T480A, D508G, L510F, Y533C, V540D) have been lost from the shuffled variants. The single exception to this is the G81V substitution that has survived in the Shuffled 2 variant. S557 has been converted to proline in both cases, again the best substitution identified at this position. Two further substitutions have been introduced to each of the shuffled variants. Both have an additional arginine (E378R for Shuffled 1, Q498R for Shuffled 2) and interestingly both have obtained the K568Q substitution, the final substitution identified by Matsumura and Ellington but missing from our neutral libraries as it is deleterious for glucuronidase activity. The kcat/Km values obtained for both of these clones using p nitrophenyl β-D-galactopyranoside as a substrate were similar to that obtained for the mutant T509A/S557P/N566S/K568Q. Shuffling of the neutral libraries themselves failed to generate colonies exhibiting more galactosidase activity than negative controls.

### Additional Screening

The purified plasmid libraries obtained from rounds four and five of neutral drift experiment A and round four of neutral drift experiment B were transformed into BW25141 cells and the transformants were applied to LB amp plates containing 100 μg.mL^-1 ^ampicillin, 2 mg.mL^-1 ^arabinose and one of the substrates; 5-bromo-4-chloro-3-indolyl-β-Dfucopyranoside (X-fucopyranoside), 5-bromo-4-chloro-3-indolyl-β-Dxylanopyranoside (X-xylopyranoside), 5-bromo-4-chloro-3-indolyl-N-acetyl-β-Dglucosaminide (X-glucosaminide), 5-bromo-4-chloro-3-indolyl-N-acetyl-β-Dgalactosaminide (X-galactosaminide) or 5-bromo-4-chloro-3-indolyl-β-D11 glucopyranoside (X-glucopyranoside). Screening wild type β-glucuronidase against these substrates showed that it was active towards glucopyranoside and weakly active towards xylopyranoside but inactive towards fucopyranoside, glucosaminide and galactosaminide. Three colonies in the round four library from neutral drift experiment A appeared blue compared to the other colonies in the screened library and compared to wild type β glucuronidase colonies when screened against the substrate X-xylopyranoside. Three unique gene sequences were identified; F88S/I170V/A323T, Q385L/F448H/T509A and Q40R/A560P/N574S/G594R/E595D. Geddie and Matsumura [19] used site saturation mutagenesis of the active site loop residues 557, 566 and 568 to produce clones exhibiting increased xylosidase activity. T509A is the only mutation the two studies have in common. Therefore, our neutral selection experiment has identified other residues that may contribute to xylosidase activity.

## Discussion

A central problem with many directed evolution experiments based on random mutation is the tendency for the selection process to become trapped in a local minimum. The original experiments of Matsumura and Ellington [13] on beta-glucuronidase exemplify this with their final selected variant, while performing orders of magnitude better in the target activity than the original protein, still performing an order of magnitude worse than that of a naturally evolved beta-galactosidase. Much of the technical development of new methods for creating libraries for directed evolution has been focussed on developing methods and protocols to overcome this problem via saturation mutagenesis, recombination, or other approaches. The use of neutral selection appears to offer a complementary approach to avoiding the problem of local minima by increasing the spread of starting points in the "fitness landscape". If the problem of the conventional approach is that it only allows variants to find the top of the hill on which the researcher begins, then neutral selection offers a way of increasing the range of those starting points while remaining in "functional areas". This avoids the problems that saturation mutagenesis can cause by generating a large proportion of non-functional variants for screening.

Thus the aim of our experiments was to use neutral selection to identify new variants that can provide improvements to the desired function that are not accessible by a conventional direct selection experiment based on random selection. From this perspective the results are disappointing as we have identified only variants and positions of mutation that have been described in other direct selection experiments aimed at converting β-glucuronidase into a galactosidase. However, it is worth noting that by performing neutral selection we obtained improved variants with modifications at four out of the five sites previously identified. The fifth mutation which is beneficial to galactosidase activity but deleterious to glucuronidase activity was not obtained from the neutral selection, as expected, but was selected in a single round when five neutral variants were shuffled together.

When we examine other studies using neutral selection a very similar picture emerges. The neutral drift study of Gupta and Tawfik [20] produced mutational compositions similar to those obtained by directed evolution of PON 1. The best variant in the neutral drift study contained the active site mutations S193T and T332S. Mutations at these sites were previously identified in traditional directed evolution experiments [21,22]. One of the neutral mutations seen in variants improved for DEPCyC, F222S (Gupta & Tawfik, [20]) under neutral selection was not seen in direct selection for the same substrate [21]. However this was not one of the most active variants produced. Bershtein *et al*. [9] carried out an accelerated neutral drift experiment using TEM-1 β lactamase producing a range of mutations that increased protein stability. The most common enriched mutations found in the study were previously identified when a destabilized form of TEM-1 was evolved to regain stability [23]. Mutations at the same sites as the remaining enriched mutations and all but three of the purging mutations were also previously generated by Bershtein *et al*. [24] when they subjected TEM-1 to random mutational drift and purifying selection (to purge deleterious mutations). Therefore, the mutations found in the neutral drift studies of Gupta and Tawfik [20], and Bershtein *et al*. [9] produced variants with mutations at the same positions as those produced from more conventional evolution experiments on the same proteins.

It is possible that clonal interference may prevent certain mutations from appearing. Rowe *et al*. [15] compared directed evolution approaches using β glucuronidase and found that the beneficial mutations (F365S/W529L) could drive N566S into extinction even though the mutations were potentially synergistic in effect. They demonstrated that the undesirable extinction events could be prevented by DNA shuffling. The mutation K568Q did not appear in our neutral drift library because of the loss of original glucuronidase activity. However, DNA shuffling of our neutral clones produced galactosidase positive variants containing this mutation. Therefore DNA shuffling following either directed evolution or neutral drift experiments may overcome both clonal interference and trade off restrictions.

Overall it appears that neutral selection does not provide new and improved variants over those seen in conventional directed evolution experiments based on random mutagenesis and direct selection.

If neutral selection does not provide improved variants then it may still be of value if it identifies those variants more efficiently. This is the central claim made by Gupta and Tawfik [20]. In the case of β-glucuronidase Matsumura and Ellington undertook three rounds of selection, screening 7 000, 20 000, and 7 500 colonies in each round respectively [13]. In our experiments we screened smaller numbers; a total of between 8 000 and 17 000 colonies for four rounds of the neutral screen and 5000 to 8 500 for the galactosidase screen. Arguably this makes the neutral approach more efficient but the advantages are marginal. When we carried out an experiment using very small library sizes of less than 600 variants per round no positive variants were found when we screened for galactosidase activity (see supplementary information for details). The lack of β-galactosidase positive variants was attributed to using such a small library size.

The added complication of developing a screen for a second activity and optimising the mutation rate is enough of a disincentive that the neutral approach is unlikely to be appealing for most straightforward optimization experiments.

Our experiment was limited to relatively small numbers as the aim was to investigate the value of neutral selection in conditions similar to those carried out in most directed evolution experiments. One important question is whether the results would be different had larger numbers of variants been screened? Experiments using saturation mutagenesis of the β-glucuronidase gene have identified variants with greater increases in β-xylosidase activity than the increase in β-galactosidase activity previously observed via random mutagenesis [19]. However the only one of these characterised for glucuronidase activity has a massive decrease in kcat. A larger scale screen using assays with much higher throughput, such as FACS analysis [25], would be required to investigate this more fully. Again the studies of Gupta and Tawfik using serum paraoxonase [20] and Bloom *et al*. [8] using cytochrome P450 do not identify new variants despite screening much larger numbers. Our conclusion, based on the combination of these studies and are own, is that there is little to be gained by increasing screening sizes from thousands to millions for neutral selection. If it is feasible to screen very large numbers then direct selection is likely to generate the same results with a similar amount of effort.

**Figure 3:**
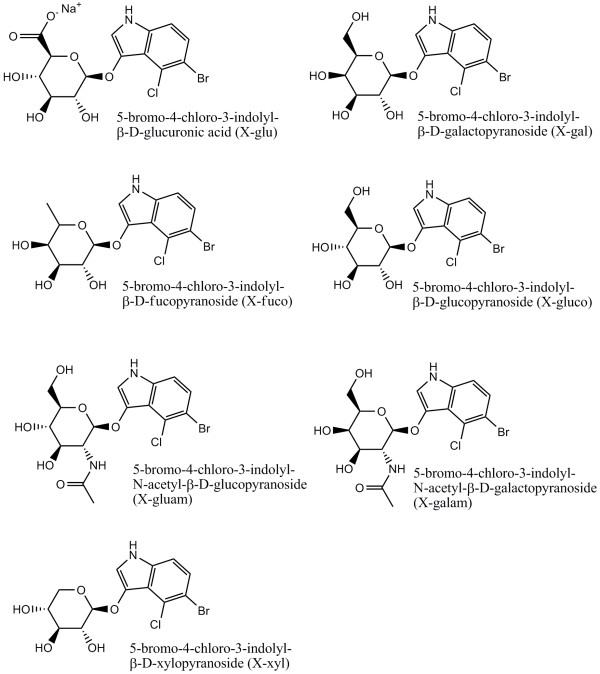
**Structures of the alternative substrates**. Structures of the substrates 5-bromo-4-chloro-3-indolyl-β-D-fucopyranoside (X-fucopyranoside), 5-bromo-4-chloro-3-indolyl-β-D-xylanopyranoside, (X-xylopyranoside) 5-bromo-4-chloro-3-indolyl-N-acetyl-β-D-glucosaminide (X-glucosaminide), 5-bromo-4-chloro-3-indolyl-N-acetyl-β-D-galactosaminide (X-galactosaminide) and 5-bromo-4-chloro-3-indolyl-β-D-glucopyranoside (X-glucopyranoside) used in the additional screening of the fourth round neutral drift libraries.

Given that the neutral selection process creates a library capable of generating useful variants for a modified function after one further round of direct selection, an area where it may find useful application is where a library is desired for generating multiple, related functions. We demonstrated this by screening our libraries against a range of x-glycosides and identifying specific improved variants. The chosen glycosides (Figure 3) displayed a range of structural changes compared to glucuronides. As expected the neutral library had not developed activity towards β-fucosides, β-glucosaminides or β-galactosaminides. Three variants, F88S/I170V/A323T, Q385L/F448H/T509A and Q40R/A560P/N574S/G594R/E595D, were found to have increased activity towards β-xylopyranosides compared to wild type β- glucuronidase. Again this was achieved in only one round of selection. In this T509A is the only mutation in common with the experiment carried out by Geddie and Matsumura [19] in which saturation mutagenesis was used to direct the evolution of GUS variants with increased xylosidase activity. However we have no reason for thinking that the other variants we identify would not be found in a conventional directed evolution experiment.

Matsumura and Ellington [13] screened a selection of mutants found during their experiment for glucosidase and fucosidase activity. Their most efficient mutant for β-galactosidase activity, T509A/S557P/N566S/K568Q, was also found to have developed increased activity towards fucosides and glucosides compared to wild type β-glucuronidase. Likewise the single mutant N566S and the quadruple mutant T509A/D531E/S557P/N566S had increased fucosidase activity.

Neutral selection in the case reported here performs no better than several rounds of direct selection but can provide a library containing variants that perform against a variety of substrates that can be selected in a single round of direct selection. If multiple similar activities are desired then neutral selection can be an efficient route to them. Overall the results we present here do not provide evidence that supports the use of neutral approaches as a more efficient approach to directed evolution. At least for the system we have investigated the additional diversity potentially provided by this approach is either insufficient or of the wrong type to provide access to local selection optima different from those found in direct selection experiments. This may of course vary from system to system and a detailed statistical study of a wide range of systems where different selection protocols to libraries of varying diversity seems a promising course for future study.

## Materials and methods

See [Additional file 3].

## Competing interests

The authors declare that they have no competing interests.

## Authors' contributions

WSS carried out the initial neutral drift experiments, the characterisation of neutral and positive variants, the DNA shuffling experiments and helped to draft the manuscript. JRH carried out the later neutral drift experiments, the additional substrate screening and contributed to the drafting of the manuscript. CN conceived the study, participated in its design and coordination and drafted the manuscript. All authors have read and approve the final manuscript.

## Supplementary Material

Additional file 1 **Materials and Methods**. Full description of materials and methods used. Click here for file

Additional file 2 **Supplementary figure S1**. a set of three pictures demonstrating the colour changes observed in bacterial colonies exhibiting a positive reaction for X-glu and X-gal. Click here for file

Additional file 3 **Supplementary Table T1**. a table detailing the numbers of colonies counted at each stage in each library. Click here for file
