# Supplementary material for: Applying neutral drift to the directed molecular evolution of a β-glucuronidase into a β-galactosidase: Two different evolutionary pathways lead to the same variant
Source: BMC Res Notes. 2011 May 6;4:138. doi: 10.1186/1756-0500-4-138 (PMC3118342; doi:10.1186/1756-0500-4-138)
Supplement: Additional file 1 — Materials and Methods. Full description of materials and methods used. [file 1756-0500-4-138-S1.DOC]

T509A, S557P, N566S, K568Q
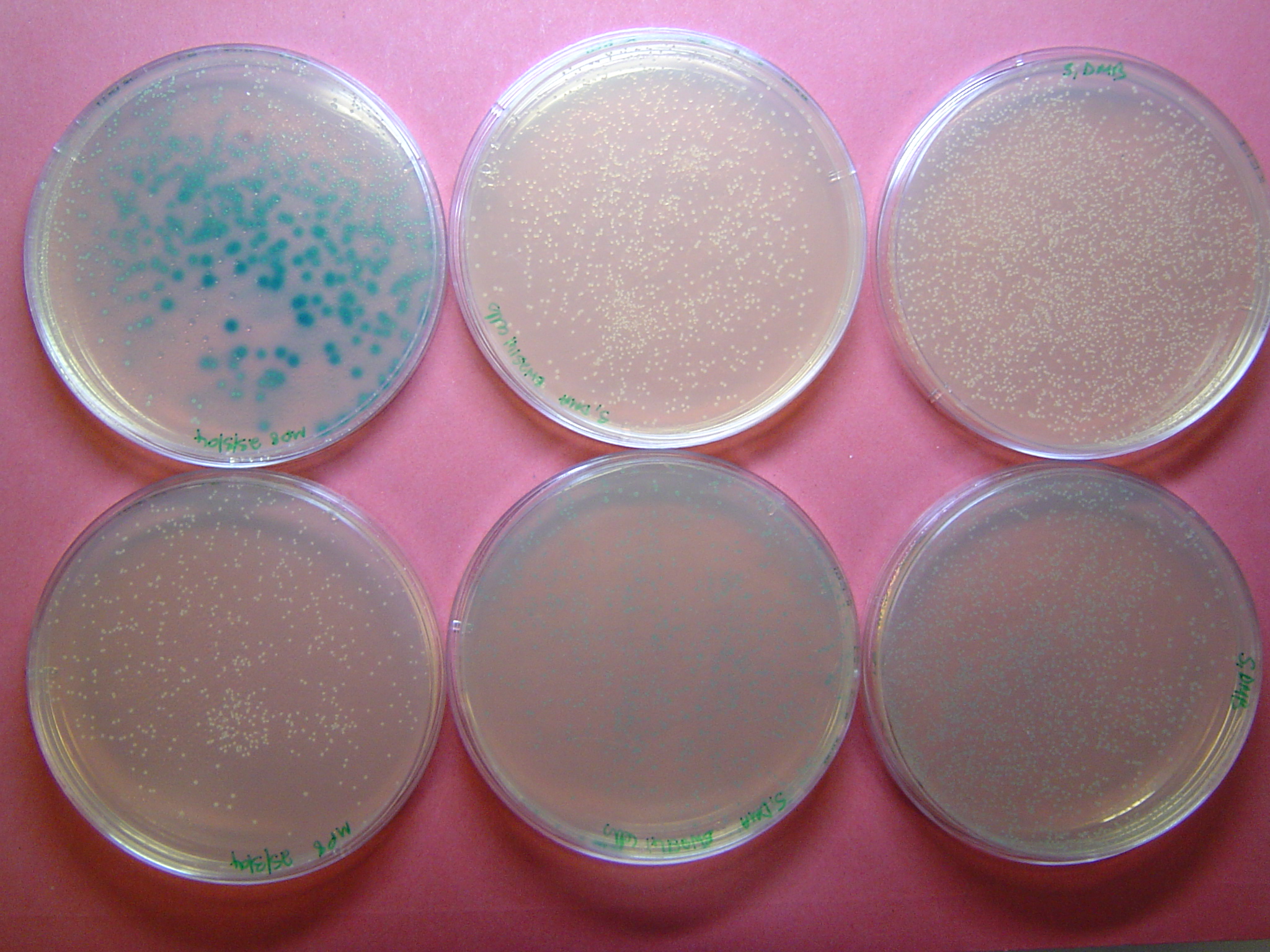


X glu

X gal

β glucuronidase S557P, N566S, K568Q

1. Examples of x-glu and x-gal screening for wild type β glucuronidase and the mutants S557P/N566S/K568Q and T509A/S557P/N566S/K568Q.


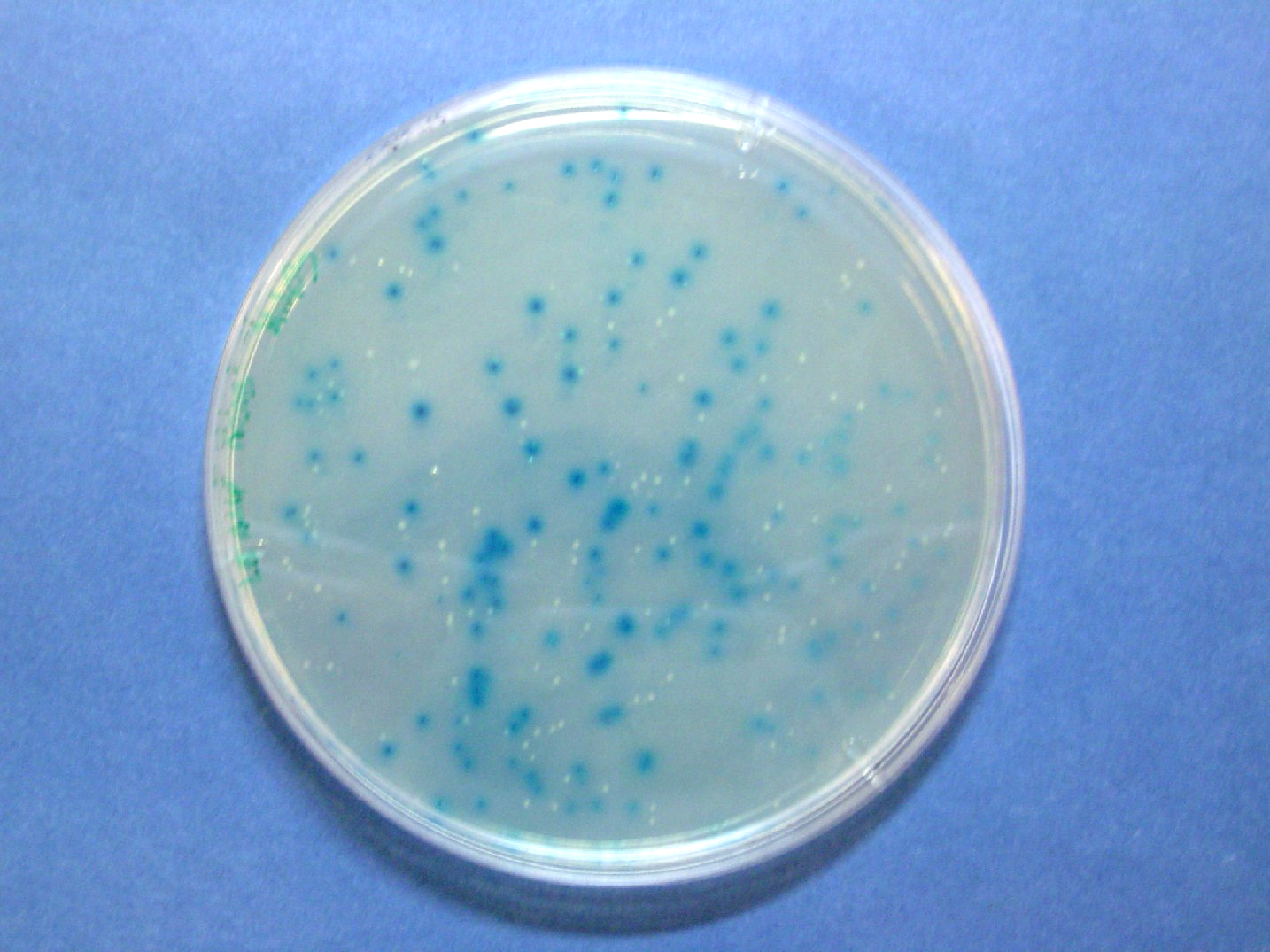


1. Example x-glu screening of round three library.


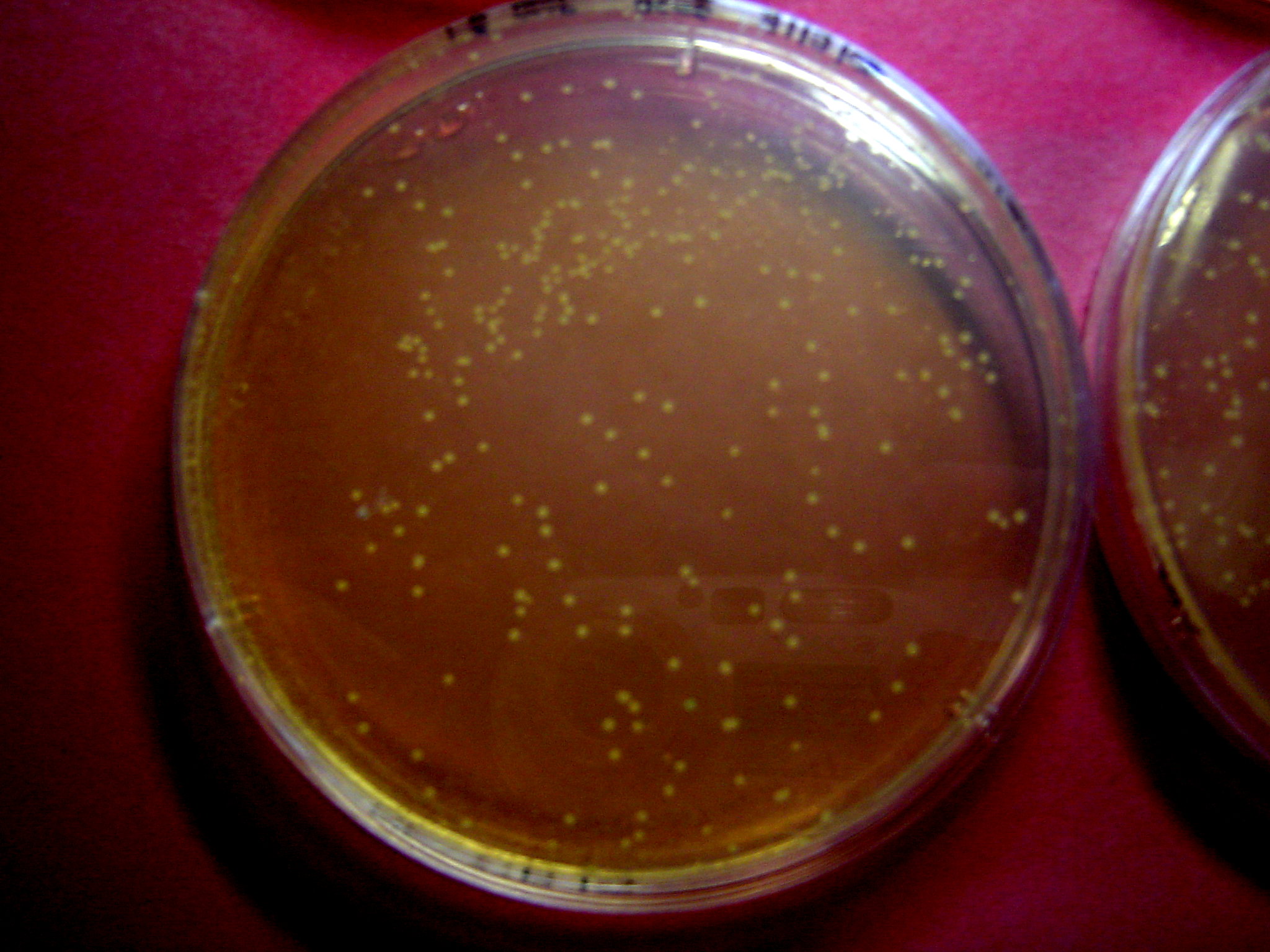


1. X-gal screening of round four library.

Supplementary Figure 1: Examples of plate screening using x-glu and x-gal as substrates.
