# Supplementary material for: Applying neutral drift to the directed molecular evolution of a β-glucuronidase into a β-galactosidase: Two different evolutionary pathways lead to the same variant
Source: BMC Res Notes. 2011 May 6;4:138. doi: 10.1186/1756-0500-4-138 (PMC3118342; doi:10.1186/1756-0500-4-138)
Supplement: Additional file 2 — Supplementary figure S1. a set of three pictures demonstrating the colour changes observed in bacterial colonies exhibiting a positive reaction for X-glu and X-gal. [file 1756-0500-4-138-S2.DOC]

| Round number | XL1 Blue colony numbers | Blue colonies  (X-glu screen) | White colonies  (x-glu screen) | Total number of colonies | % blue colonies | Number of colonies screened (x-gal) |
| --- | --- | --- | --- | --- | --- | --- |
| Experiment A |  |  |  |  |  |  |
| 1 | 15033 | 1815 | 711 | 2526 | 72 |  |
| 2 | 10263 | 801 | 343 | 1144 | 70 |  |
| 3 | 11376 | 1341 | 762 | 2103 | 64 |  |
| 4 | 6837 | 952 | 634 | 1586 | 60 | 6000 |
| 5 | 6524 | 340 | 312 | 652 | 52 | 2514 |
| Experiment B |  |  |  |  |  |  |
| 1 | 8506 | 971 | 794 | 1765 | 55 |  |
| 2 | 7563 | 1706 | 1848 | 3554 | 48 |  |
| 3 | 9501 | 2204 | 1663 | 3867 | 57 |  |
| 4 |  |  |  |  |  | 5263 |
|  |  |  |  |  |  |  |
| Experiment C |  |  |  |  |  |  |
| 1 | 1533 | 2825 | 899 | 3724 | 75 |  |
| 2 | 5743 | 3064 | 424 | 3488 | 87 |  |
| 3 | 1788 | 3262 | 2372 | 5634 | 58 |  |
| 4 | 1382 | 1902 | 2735 | 4637 | 41 | 6000 |
|  |  |  |  |  |  |  |

Supplementary Table 1: Table of colony numbers produced during each round of mutagenesis for experiments A, B and C.
