# Supplementary material for: Applying neutral drift to the directed molecular evolution of a β-glucuronidase into a β-galactosidase: Two different evolutionary pathways lead to the same variant
Source: BMC Res Notes. 2011 May 6;4:138. doi: 10.1186/1756-0500-4-138 (PMC3118342; doi:10.1186/1756-0500-4-138)
Supplement: Additional file 3 — Supplementary Table T1. a table detailing the numbers of colonies counted at each stage in each library. [file 1756-0500-4-138-S3.DOC]

**5 Materials and Methods**

**5.1 Materials**

Genemorph™ and Genemorph II™ Random Mutagenesis Kits and XL1-Blue supercompetent cells were from Stratagene. T4 DNA ligase, *Taq* polymerase and the Wizard Plus SV DNA purification system were obtained from Promega. *Nco*I and Vent DNA polymerase were obtained from New England Biolabs and *Eco*RI from Roche*.* dNTPs were purchased from Bioline. 5-bromo-4-chloro3indolyl-β-D glucuronic acid sodium salt (x-glu) was obtained from Acros Organics; 5 bromo-4chloro-3-indolyl-β-D-galactoside (x-gal) was obtained from Qbiogene. 5-bromo-4-chloro-3-indolyl-β-D-fucopyranoside, 5-bromo-4-chloro-3-indolyl-β-D-xylanopyranoside, 5-bromo-4-chloro-3-indolyl-N-acetyl-β-Dglucosaminide,5-bromo-4-chloro-3-indolyl-N-acetyl-β-D-galactosaminide and 5-bromo-4-chloro-3-indolyl-β-D-glucopyranoside were purchased from Fluka. PNP-glu and PNP-gal were from Sigma.

**5.2 Cloning of** β−**glucuronidase**

The *gusA* gene was amplified from *E. coli* K12 genomic DNA using Vent polymerase and the primers 5’-ACGTCGCCCATGGGACACCATCACCATCACCATATGTTACGTCCTGTAGAAACC (OSOT165) and 5’-CGCGAATTCTGCAGTCATTGTTTGCCTCCCTGCT (OSOT166). The PCR product was digested with *Nco*I and *Eco*RI and ligated into pBAD/His to generate the plasmid pWSS042. The *gusA* plasmid was transformed into XL1-Blue and ampicillin resistant transformants were selected. The DNA sequence of the *gusA* gene was confirmed by sequencing.

**5.3 Random mutagenesis**

The β-glucuronidase gene was mutated randomly using the Stratagene Genemorph mutagenesis kit or via error prone mutagenesis with *Taq* polymerase. Genemorph reactions contained the amplification primers OSOT165 (0.2μM) and OSOT166 (0.2μM), 0.2mM dNTPs and the supplied buffer and were thermally cycled 30 times through 94^o^C for 1 minute, 50^o^C for 1 minute and 72^o^C for 2 minutes. Error-prone PCR reactions included OSOT165, OSOT166, 62.5 μM MnCl2, 0.5 mM MgCl2, 10mM Tris HCl, 50mM KCl, 0.1% TritonX-100 and mutagenic dNTP mix (1 mM dCTP and dTTP, 0.2 mM dGTP and dATP). The reactions were thermally cycled 30 times through 95^o^C for 1 minute, 50^o^C for 1 minute and 72^o^C for 2 minutes. The PCR products were digested with *Nco*I and *Eco*RI and ligated into pBAD/His to generate the initial library.

**5.4 Plate assays for glucuronidase and galactosidase activity**

β-glucuronidase activity was detected using 5-bromo-4-chloro-3-indolyl-β-D glucuronic acid

sodium salt (x-glu). The library of *gusA* genes in pBAD/His was transformed into XL1-Blue super competent cells. Transformants were applied to LB agar plates containing 100 μg.mL^-1^ ampicillin. All of the resulting colonies (between 6 000 and 15 000) were re-suspended in 10 mL of LB medium and plasmid DNA was isolated. The purified plasmid library was then transformed into electrocompetent BW25141 [14] by electroporation. Transformants were applied to LB agar plates containing 100 μg.mL^-1^ ampicillin and 2 mg.mL-1 arabinose. Glucuronidase substrate (40 μL of a 20 mg.mL^-1^ solution in DMSO) was spread onto the plates one to two hours before use. Blue colonies, indicating the presence of an active glucuronidase activity, were selected and re-suspended in LB medium, and plasmid DNA was purified directly from the cell suspension without further growth. Further rounds of mutagenesis, cloning and screening were carried out in the same way. The number of colonies screened on x-glu agar plates ranged from 800 to 3 000. β-galactosidase activity was determined using the same expression system and saturating concentrations of 5 bromo-4-chloro-3-indolyl-β-D-galactoside (x-gal). The sensitivity of the detection system was confirmed by creating the mutant T509A/S557P/N566S/K568Q previously identified by Matsumura and Ellington. BW25141 cells were propagated on LB agar plates containing 100 μg.mL^-1^ ampicillin and 2 mg.mL^-1^ arabinose. The plates were coated with 400 μl of a 20 mg.mL-1 solution of x-gal in DMSO two to three hours prior to use.

**5.5 Plate assays for fucosidase, glucosaminidase, galactosaminidase, glucosidase and xylosidase activity**

Purified plasmid libraries were transformed into electrocompetent BW25141by electroporation. Transformants were applied to LB agar plates containing 100μg.mL^-1^ ampicillin and 2 mg.mL^-1^ arabinose. Plates were prepared with one of each of the substrates 5-bromo-4-chloro-3-indolyl-β-D-fucopyranoside (X-fucopyranoside), 5-bromo-4-chloro-3- indolyl-β-D-xylanopyranoside, (X-xylopyranoside) 5-bromo-4-chloro-3-indolyl-N-acetyl-β- D-glucosaminide (X-glucosaminide), 5-bromo-4-chloro-3-indolyl-N-acetyl-β-Dgalactosaminide (X-galactosaminide) and 5-bromo-4-chloro-3-indolyl-β-D-glucopyranoside (X-glucopyranoside) (400 μL of a 20 mg.mL^-1^ solution in DMSO) one to two hours before use.

**5.6 Site directed mutagenesis**

Site directed mutagenesis was carried out using a Stratagene site directed mutagenesis kit.

The primers used were:

N566S/K568Q 5’-CGTTGGCGGTAGCAAGCAAGCAAGGGATCTTCAC-3’ and 5’- GTGAAGATCCCTTGCTACCGCCAAGC-3’,

S577P 5’-GATTTTGCGACCCCGCAAGGCATATTG-3’ and 5’- CAATATGCCTTGCGGGGTCGCAAAATC-3’,

T509A 5’-CGAATACGGCGTGGATGCGTTAGCCGGGCTGC-3’ and 5’- GCAGCCCGGCTAACGCATCCACGCCGTATTCG-3’,

L510F 5’-GAGTGCAGCCCGGCAAACGTATCCACGCCG-3’ and 5’- CGGCGTGGATACGTTTGCCGGGCTGCACTC-3’,

N566S 5’-GAAGATCCCTTTCTTGCTACCGCCAACGCGC-3’ and 5’- GCGCGTTGGCGGTAGCAAGAAAGGGATCTTC-3’,

D508G 5’-CGAATACGGCGTGGGTACGTTAGCCGG-3’ and 5’- CCGGCTAACGTACCCACGCCGTATTCG-3’,

K568Q 5’-GGCGGTAACAAGCAAGGGATCTTCACTC-3’ and 5’- GAGTGAAGATCCCTTGCTTGTTACCGCC-3’.

**5.7 DNA sequencing**

DNA sequencing was carried out by MWG Biotech or in house using a Beckman Coulter CEQ8000 system. The sequencing primers used were 5’-CGATTAAAGAGCTGATAGCG- 3’, 5’-GCTTTGGTCGTCATGAAGATGC-3’, 5’- CCGTTTGTGTGAACAACG-3’ and 5’- ATGTCATAGCATTTTTATCC-3’.

**5.8 Enzyme purification**

Wild type β-glucuronidase and selected mutants with β-galactosidase activity were over expressed in BW25141 cells. Cells were grown in LB medium, containing 100 μg.mL^-1^ ampicillin, at 37 ^o^C to mid log phase (A600~0.6). Arabinose (final concentration 0.2% w/v) was added to induce protein expression and the cells were incubated for a further three hours.

Cells were harvested by centrifugation at 7 800g and the cell pellets stored at -80^o^C until required. Cell pellets were re-suspended in 50 mM Tris HCl pH 8, 10% glycerol (4 mL.g-^1^ wet weight of cells) and lysed by sonication. The solution was centrifuged at 23 400g for 30 minutes at 4^o^C. The cleared lysate was applied to a Ni-NTA sepharose FF column equilibrated in 50 mM Tris HCl pH 7.4. The column was washed with Buffer A (50 mM Tris-HCl pH 7.4, 0.5 M NaCl, 60 mM imidazole) and the protein eluted in Buffer B (50 mM Tris HCl pH 7.4, 0.5 M NaCl, 0.5 M imidazole). The protein was concentrated and exchanged into 50 mM Tris HCl pH 7.4 using an Amicon ultra filtration cell. Alternatively, protein was purified from the cleared lysate via FPLC using a Ni-NTA sepharose column equilibrated and washed in Buffer A until all unwanted fractions were eluted. The protein was eluted in Buffer B and exchanged into 50 mM Tris HCl pH 7.4 using a PD10 column. Activity assays were carried out immediately. However, the proteins retained their activity after storage in 10% glycerol, 50 mM Tris HCl pH 7.4 at -20^o^C.

**5.9 Solution assays of glucuronidase and galactosidase activity**

The substrates *p*-nitrophenyl-β-D-galactopyranoside (pNP-gal) and *p*-nitrophenyl-β-Dglucuronide (pNP-glu) were diluted in 50 mM Tris HCl buffer pH 7.4 such that 50 μl aliquots could be added to a total reaction volume of 100 μl and give the desired substrate concentration. Aliquots were added to a 96 well plate and the reaction was initiated by the addition of protein. The absorbance at 405 nm was monitored using a Safire2 microplate reader (Tecan). An extinction coefficient of 17 400 M^-1^ cm^-1^ was used to calculate *p*-nitrophenyl- phosphate concentration. The concentration of protein used ranged from 0 to 200 nM in the glucuronidase assays and from 500 nM to 5 μM in the galactosidase assays. The concentration of pNP-gal ranged from 100 μM to 15 mM and that of pNP-glu varied between 10 μM and 5 mM.

**5.10 DNA shuffling**

DNA shuffling was carried out using the method of Stemmer [17, 18]. PCR products were digested with DNase I, in the presence of 1.25 mM MgCl2 and 50 mM Tris HCl pH 7.4, at room temperature for 10 minutes. The samples were heated to 96^o^C for 20 minutes, placed on ice then loaded onto a 2% low melting point gel. The fragments from 50-200 base pairs were excised from the gel and purified using the Promega Wizard DNA purification system. The purified products were then subjected to thermal cycling without primers. The reactions contained 0.2 mM dNTPs, 10 mM Tris HCl, 50 mM KCl, 0.1% TritonX-100, 2 mM MgCl2, purified digested product and Taq polymerase. Thermal cycling was carried out through 94^o^C for 30s, 55^o^C for 30s and 72^o^C for 1 minute for 39 cycles. The products of the first thermal cycling reaction were then subjected to a second round of thermal cycling in the presence of primers. The reactions were thermally cycled ten times through 94^o^C for 1 minute, 40^o^C for 1 minute, 72^o^C for 5 minutes followed by 30 cycles of 94^o^C for 1 minute, 50^o^C for 1 minute, 72^o^C for 5 minutes. The resulting PCR products were digested with *Eco*RI and *Nco*I, ligated into pBAD/His and transformed in XL1-Blue super competent cells for screening.
